# Brexit and Trump: Which Theory of Social Stasis and Social Change Copes Best With the New Populism?

**DOI:** 10.3389/fpsyg.2022.797139

**Published:** 2022-05-26

**Authors:** Chuma Kevin Owuamalam, Mark Rubin, Russell Spears

**Affiliations:** ^1^Division of Organizational and Applied Psychology, University of Nottingham Malaysia, Semenyih, Malaysia; ^2^Department of Psychology, Durham University, Durham, United Kingdom; ^3^Department of Social Psychology, University of Groningen, Groningen, Netherlands

**Keywords:** Brexit and Trump, social identity, SIMSA, system justification, disadvantage, voter attitudes

## Abstract

Why do voters seek to change the political landscape or to retain it? System justification theory (SJT) proposes that a separate system motive to *preserve* the existing order drives support for the status-quo, and that this motivation operates independently from personal and collective interests. But how does this explanation apply to recent populist shifts in the political order such as Brexit and the emergence of Donald Trump? While the system motive may seem useful in understanding why the usual progressives (Remain/Clinton voters) may want to stick with an established order, it seems insufficient to explain why the more conservative voters (Brexit/Trump voters) would want to upend the establishment. Thus, we compared SJT’s system motive explanation for the system attitudes of voters on both sides of the political divide to an alternative explanation drawn from the newer social identity model of system attitudes (SIMSA). According to SIMSA, the difficulty in explaining the system attitudes of Brexit/Trump and Remain/Clinton voters from SJT’s system motive standpoint can be resolved by focusing instead on the *collective interests* that both camps seek to satisfy with their votes. We examined these explanations in two studies conducted soon after Brexit (*N* = 313) and Trump’s election (*N* = 289) in 2016, with results providing more support for SIMSA than for SJT.

## Introduction

There has been a growing anti-establishment populism in Europe and North America (United States), of which Brexit and Donald Trump’s election to the office of the US president are two prominent examples. In these examples, the populists have clamoured for a change to the status quo vis-à-vis the United Kingdom exiting the European Union (EU; Brexit) or the election of a non-politician, anti-establishment member of a nonincumbent political party to power (Donald Trump). In contrast, opponents of these movements have sought to maintain the existing social order by retaining the United Kingdom’s membership of the EU or nominating a professional politician who is a member of the incumbent political party and the Washington establishment (Hillary Clinton). In the present paper, we referred to these two political developments in order to examine how prominent social psychological theories account for social stasis and change. Specifically, we investigated explanations derived from system justification theory (SJT; Jost and Banaji, 1994) and the social identity model of system attitudes (SIMSA; Owuamalam et al., 2018, 2019a,b) regarding the motives underlying people’s political attitudes and voting preferences.

System justification theory (Jost and Banaji, 1994) proposes that a fundamental *system justification motive* helps to explain why people might want to cling on to existing systems. Central to SJT is the idea that people have an inherent need to preserve societal hierarchies “even at considerable cost to themselves and to fellow group members” (Jost and Hunyady, 2005, p. 260). According to SJT, threats to the status quo can often bring about uncertainties, and the resulting fear and anxiety could, in turn, undermine people’s control over their life’s outcomes (see Jost and Hunyady, 2005; Table 1). Hence, people (e.g., British EU referendum and US election voters) may be motivated to *rationalize* the status quo in order to escape uncertainty and to maintain control over their lives.

**Table 1:** Zero-order bivariate correlation between mechanisms related to system justification and personal/collective interests.

	1	2	3	4	5	6	7	8
1. Collective interest	−	0.32^***^	0.28^***^	−0.20^***^	0.30^***^	0.03	0.19^***^	
2. Personal interest	0.41^***^	−	0.30^***^	−0.06	0.27^***^	−0.03	0.12^*^	
3. Uncertainty avoidance	0.24^***^	0.37^***^	−	0.04	0.10	−0.06	0.01	
4. Fear	−0.09	0.16^*^	0.17^*^	−	−0.03	0.03	0.02	
5. Control maintenance	0.37^***^	0.50^***^	0.38^***^	0.14^*^	−	0.01	0.11+	
6. Social class (household income)	−0.07	0.06	0.09	0.02	0.09	−	0.06	
7. Personal sense of power	0.16^**^	0.18^**^	0.08	−0.03	0.12^*^	0.22^***^	−	
8. To preserve the establishment	0.07	0.03	0.01	0.25^***^	0.11	0.06	−0.03	−
9. Confidence in the system	−0.03	0.02	−0.02	0.17^**^	0.08	0.06	0.04	0.73^***^

* *p* < 0.05;

** *p* < 0.01;

*** *p* < 0.001.

Coefficients in the upper diagonal of the correlation matrix relate to Study 1, while those in the lower diagonal relate to Study 2.

However, much of the theoretical debate between system justification theorists (e.g., Jost et al., 2004, 2019; Jost, 2019, 2020) and social identity theorists (Spears et al., 2001; Reicher, 2004; Rubin and Hewstone, 2004; Owuamalam et al., 2019a,b) has centered on whether a system justification motive is *necessary* to explain cases of system support or change, especially among people who might be disadvantaged by the established political system. Social identity theorists have suggested that a separate system justification motive may not be necessary to explain system justification-like attitudes, and that rationalization of the status quo may be more parsimoniously explained as either (a) a passive acceptance of the social realities of the intergroup context (Spears et al., 2001; Rubin and Hewstone, 2004), (b) a form of ingroup bias expressed at a superordinate level of self-categorization (e.g., Afro-Americans may support American systems when their collective American identity is salient; Caricati et al., 2021), or (c) an identity-management strategy in which the system is supported in the hope that it will eventually yield benefits for the ingroup Owuamalam et al., 2016a, 2017, 2021; Bonetti et al., 2021; Carvalho et al., 2021). We refer to this family of social identity explanations as the social identity model of system attitudes (SIMSA; Owuamalam et al., 2018, 2019a,b). Crucial to SIMSA is the idea that group-interests drive attitudes toward both stasis or change: People support the established order or want to change it because of their identification with relevant social groups (see also Caricati and Sollami, 2017). Our central question is how well SJT and SIMSA can be applied to understanding the political populism represented by Brexit and Trump, in the context of a change that seems driven by “reactionary radicalism”?

## The New Political Populism

By *the new political populism* we mean recent social movements, epitomized by Brexit in the United Kingdom and the election of Trump in the United States in 2016, in which a backlash against the prevailing political establishment seemed apparent. This populism is typically right-wing (i.e., more conservative-leaning) rather than progressive/liberal, and it can also be seen in authoritarian and anti-immigration shifts in other European countries (e.g., Germany, Hungary, Turkey, etc.).

We argue that this new political context raises a problem for SJT. SJT equates resistance to change as support for the “system,” which is typically seen as the established social order. However, because the new populism in some respects goes against the prevailing political order, and is mostly championed by people on the political right, it places SJT in contrast to the impetus for this populism because it promotes radical (political) change rather than support for the existing establishment. On the other hand, because this populism is typically conservative in character, it also arguably harks back to an even older and more established political order and its associated values. For example, Brexit involved a nostalgia for a lost political sovereignty that might be regained, and Trump’s election reflected the reassertion of America internationally and the largely white working- and middle-class at home. Common nationalist and anti-immigration themes thus define this radical but reactionary agenda. In short, it is not clear how SJT orients to this more ambiguous political landscape despite a recent foray into this topic (Azevedo et al., 2017). In particular, it is unclear how the typical system justification motives might predict political attitudes in this context. Where exactly do we locate “the system” that might be justified in this context? This question is especially important in light of the operational dogma of the system justification motive, defined as a force that propels people to support “existing” arrangements in their society, otherwise referred to as the status-quo (Jost and Banaji, 1994, p. 2; Jost et al., 2004, 2017, p. 883, p. 74; Jost, 2019, p. 263, 265, 266). Thus, while we can extrapolate some principles around support for stasis vs. change from SJT, we may need to be circumspect about what to predict on the basis of SJT in this new and ambiguous context.

In contrast to SJT, SIMSA is more grounded in the intergroup analysis of social identity theory (Tajfel and Turner, 1979), which emphasises collective interests while being less dependent on defining “the system.” This emphasis on “the group” rather than “the system” makes prediction in this political context arguably less problematic. Specifically, SIMSA predicts that political motives will typically reflect the group identities and interests of the camps involved without a separate motivation to defend the status quo, or system as such, however this is defined by SJT.

In the present research, we *explored* whether each theoretical framework (SJT and SIMSA) could account for political attitudes in this specific political context. We measured the relevant motives, including uncertainty avoidance, the need for control, and the pursuit of group interests. We then examined which motive(s) were most predictive in the Brexit and Trump contexts.

One of the central assumptions of SJT is that a system justification motive is most visibly demonstrated among the disadvantaged because supporting the status quo is oppositional to their personal and group interests. Hence, we also measured participants’ social class as an index of advantage and disadvantage (Jost et al., 2004, p. 887; see also Brandt, 2013; Brandt et al., 2020). Following SJT, the salience and/or strength of collective interests should be less prominent reasons for system support among those at the lower rung of the social class ladder. In contrast, for SIMSA, collective interests are key predictors of system attitudes, even among members of disadvantaged groups. Note that, following past research, we included both subjective measures of social class (based on a sense of personal power; Van der Toorn et al., 2015) and objective measures (based on income band; Li et al., 2020; Buchel et al., 2021).

## Study 1: The United Kingdom’s 2016 EU Referendum

The United Kingdom is one of 28 member states of the EU. However, there has been a popular concern in the United Kingdom over the EU’s policy of free movement between borders, with perceived (or actual) pressures on social systems, such as healthcare, education, and social welfare, especially in regard to EU citizens moving from less affluent nations (e.g., Poland) to more affluent member states (e.g., the United Kingdom). However, despite the unpopularity of immigration in the United Kingdom, opinions were clearly divided about whether to leave the EU or to remain in it. Those who wanted to *leave* the EU were concerned about sovereignty and the pressures that mass immigration places on their societal systems, whereas those who wished to *remain* were concerned about escaping economic uncertainties and avoiding the loss of the benefits of EU membership.

These political conditions provided a context within which to test SJT’s propositions because the relatively high levels of system threat (Jost and Hunyady, 2005), system dependency (Kay et al., 2009), and system inevitability (Kay and Zanna, 2009) provide optimum conditions for system justification. For example, there was a clear threat to the status quo vis-à-vis (a) the possibility of detaching the United Kingdom from the EU and (b) the potential of the collapse of the EU. Also, citizens depended on their EU membership to travel freely across European borders (i.e., high system dependency). Finally, our study was conducted post-referendum when the outcome was known and a new Brexit era became inevitable. Hence, following our deductions from SJT’s system inevitability caveat, we reasoned that the motives underlying the system justification effect would be more visible among active supporters of the status quo (i.e., remain voters) in the immediate aftermath of Brexit (i.e., rendering the remain voters by this point the clearly disadvantaged group). We measured participants’ support for the new Brexit order in the current study and, based on a straightforward reading of SJT’s inevitability caveat, we anticipated that Remain voters should succumb to the inevitability of the new Brexit order and offer their support to it.

However, as we noted earlier, this reasoning can also be countered by the argument that the Leave/Brexit camp harks back to a reassertion of the system, qua political sovereignty of the United Kingdom, prior to joining the EU. This ambiguity renders the test as more *exploratory* with respect to SJT and the role of its key predictors. Indeed, the exploratory nature of the assumptions that we derived from SJT’s system inevitability caveat is also compounded by what we already know from classic (e.g., Paicheler, 1979) and contemporary (Van Bavel and Pereira, 2018) views about bipolarization and the hardening of attitudes in an opposing group context, which point to the possibility of remain voters clinging on to their preference to stick with the EU post-referendum. These ambiguities, however, do not present as much of a problem for SIMSA’s explanation for system justification because it relies on the premise that people act in their collective interests. Hence, a SIMSA-based account would predict that both sides of this political divide will see their position and/or voting preferences as being tied to their group interests.

### Method

#### Sample Size and Participants

The most nuanced analysis in our design involved the relationship between the antecedents of system justification and voter group as a function of social class (equivalent to an ANCOVA with one moderating covariate plus the interaction term). Assuming a small-to-medium effect size of *f* = 0.20 (Cafri et al., 2010) and a numerator *df* = 1, we determined from G*Power (Faul et al., 2007) that we would need 199 cases to power this analysis if we set power to 0.80 and, 265 cases if we set power to 0.90 (Cohen, 1992). We therefore aimed to recruit up to 300 participants (a) to account for unusable data and (b) to provide a more powerful test of our predictions. Data collection was completed within 2 weeks of the 2016 EU referendum. The response rate was high, with 426 attempts from members of the Prolific participant pool. However, only 313 of these attempts contained complete and usable data.^1^ To ensure quality data, we excluded participants who spent less than 5 min completing our 15–20 min survey, which also included measures that were unrelated to specific ideas that are discussed here. Participants (128 men, 185 women, *M*_age_ = 34.64 years, *SD*_age_ = 12.67 years) were mainly Whites, residing in the United Kingdom, and who voted in the 2016 EU membership referendum. They received a pro-rata payment of £5 per hour in exchange for participation. We programmed Qualtrics to collect an equal number of cases for each group of voters, and while the numbers in each group varied slightly after excluding unusable cases, this difference was negligible: *remain* (*n* = 168) and *leave* (*n* = 145) voters, *X*^2^(1) = 1.69, *p* = 0.194.

#### Materials and Procedure

Participants were first asked to indicate what their actual votes were. Specifically, participants indicated their voting record by selecting one of the following two options: “I voted to leave the EU” vs. “I voted to remain in the EU.” Participants then responded to the following motive items which we treated as single items: “I voted the way I did…” (1) “to escape economic uncertainty (i.e., uncertainty avoidance),” (2) “due to fear mongering” (i.e., fear, a state that is closely tied to existential motives under SJT framework, see Douglas et al., 2017), (3) “to maintain control over my life” (control maintenance), (4) “because my personal interest was at stake” (personal interest), and (5) “because it was in the best interest of my country” (collective interest). The first three items captured the key antecedents of the system justification motive which we examined individually. The last two items tapped personal and collective motives respectively.^2^ Participants responded to these items using a seven-point scale (1 = strongly disagree, through 4 = neither agree nor disagree, to 7 = *strongly agree*).

To validate voting preference as an indicator of behavior related to system support (vs. change), we included a three-item measure of the extent to which people trusted their government: “How much of the time do you think you can trust the government to do what is right?” (1 = none of the time, 5 = *always*); “How much of the money paid into taxes do you think the government wastes?” (1 = *waste a lot*, 5 = do not waste much); and “How many of the people running the government are crooked, in your opinion? (1 = quite a few, 5 = *hardly any*, *α* = 0.74). Assuming a vote to remain in the EU represents support for the status quo, then the trust that people have in their current government should be stronger for remain than for leave voters.

We included a measure of voters’ combined annual household income as an objective indicator of social class (Diemer et al., 2013). On this measure, participants could select whether their combined annual household income fell into one of the following income brackets: “less than £30,000,” “£30,000–£39,999,” “£40,000–£49,999,” “£50,000–£59,999,” “£60,000–£69,999,” “£70,000–£79,999,” “£80,000–£89,999,” “£90,000–£99,999,” and “£100,000 or more.” In addition, we included an eight-item measure of personal sense of power that we derived from Anderson et al. (2012); e.g., “I can get people to listen to what I say;” 1 = *strongly disagree*, 7 = *strongly agree*; *α* = 0.879. Following the principles of SJT (the strong cognitive dissonance-inspired version, Jost et al. (2003), aka the status-legitimacy thesis, Brandt, 2013), we predicted that less powerful people should be most likely to show system justification effects (see also Van der Toorn et al., 2015).

Finally, we measured participants’ post-referendum support for Brexit with a three-item scale in order to test SJT’s system-inevitability caveat: “I am pleased that Britain has voted to leave the EU”; “The EU is a failed project and I support Britain having voted to leave”; and “I would vote to leave the EU if a second referendum was presented to the public” (1 = *strongly disagree*, 6 = *strongly agree*, and *α* = 0.97).

### Results and Discussion

Table 1 depicts the bivariate correlations between the motives that were measured in this study. To confirm our assumption that a vote to remain in the EU represented greater support for the existing establishment compared to a vote to leave, we compared participants’ level of trust in their government across *leave* and *remain* voters. Consistent with SJT, this analysis revealed that remain voters reported greater confidence in their government (*M* = 2.53, *SD* = 0.83) than their Brexit counterparts (*M* = 2.15, *SD* = 0.79), *t*(311) = 4.19, *p* < 0.001, Cohen’s *d* = 0.47, *SE* = 0.12, 95% CI [0.243, 0.693].

#### Are the Antecedents of the System Justification Motive More Apparent Among Remain (Relative to Leave) Voters?

To answer this key question, we conducted independent sample *t*-tests on each motive to compare the leave and remain voters’ responses (see Table 2). Consistent with SJT, uncertainty avoidance was a significantly more prominent reason for remain voters compared to leave voters, *t*(311) = 5.90, *p* < 0.001 (see Table 2 for descriptive statistics). However, contrary to SJT, the need to maintain control over life outcomes was no more prominent for remain voters than it was for leave voters, *t*(307.73) = 0.82, *p* = 0.407, and fear was a *less* (not more) prominent reason for system supporters (remain) relative to system changers (leave), *t*(292) = 3.28, *p* = 0.001. In addition, consistent with SIMSA, collective interests were a more prominent reason for remain voters than for leave voters, *t*(262.26) = 4.51, *p* < 0.001 (see Table 1). Contrary to SJT, personal interests were also more prominent reasons for remain voters than for leave voters, *t*(311) = 6.60, *p* < 0.001. Finally, contrary to the strong cognitive dissonance-inspired version of SJT, these effects were not moderated by either income-based social class (*p*s > 0.100) or a sense of personal power (*p*s > 0.170).

**Table 2:** Reasons for System Support vs. System Change.

	Study 1: The United Kingdom’s 2016 EU Referendum			Study 2: United States’s 2016 Presidential Election			Meta-analysis
	System change (leave) voters	System support (remain) voters	Cohen’s *d* [95% CI]	System change (Trump) voters	System support (Clinton) voters	Cohen’s *d* [95% CI]	Cohen’s *d* [95% CI]
Collective interest	5.86 (1.12)	6.36 (0.83)	−0.51 [−0.739, −0.287]	5.84 (1.23)	6.13 (1.17)	−0.24 [−0.473, −0.10]	−0.38 [−0.644, −0.113]
Personal interest	4.17 (1.43)	5.30 (1.57)	−0.75 [−0.980, −0.520]	4.78 (1.54)	5.25 (1.67)	−0.29 [−0.524, −0.060]	−0.52 [−0.970, −0.073]
Uncertainty avoidance	4.37 (1.49)	5.41 (1.62)	−0.67 [−0.894, −0.438]	5.28 (1.45)	4.62 (1.69)	0.42 [0.185, 0.651]	−0.12 [−1.187, 0.938]
Fear	3.03 (1.81)	2.39 (1.62)	0.37 [0.150, 0.598]	2.54 (1.57)	3.19 (1.96)	−0.36 [−0.595, −0.130]	0.01 [−0.716, 0.728]
Control maintenance	5.30 (1.11)	5.19 (1.43)	0.09 [0.137, 0.307]	4.73 (1.44)	4.71 (1.76)	0.01[−0.218, 0.243]	0.05 [−0. 110, 0.210]
System justification motive	−	−	−	2.88 (1.52)	3.64 (1.68)	−0.47 [−0.707, −0.240]	−

Means for each voter group are presented outside parentheses, while their corresponding SDs are presented within parentheses.

#### Is SJT’s System-Inevitability Caveat Applicable in the New Political Populism?

**Figure 1:**
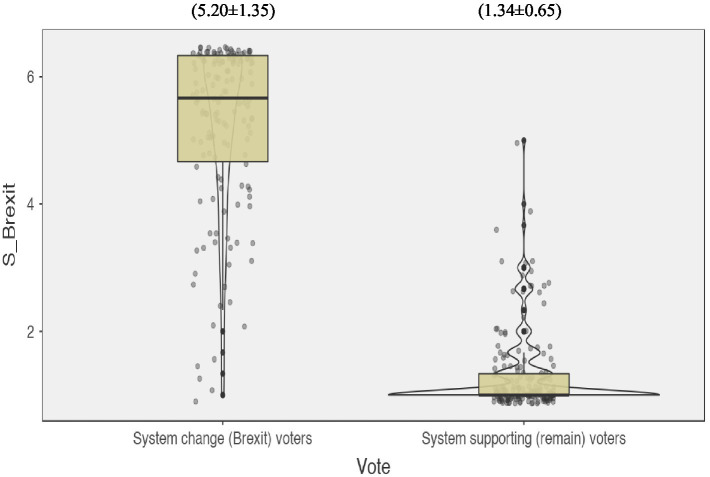
Violin plots for the distribution of scores within the system supporting vs. system change voting groups for post-referendum support for the new Brexit era. Numbers above the plots = (Mean ± *SD*). S_Brexit = support for Brexit.

Contrary to SJT’s system-inevitability caveat, results from an independent *t*-test revealed that post-referendum support for Brexit was significantly *weaker* among system supporters (remain) than among system changers (leave), *t*(200.26) = 31.38, *p* < 0.001, Cohen’s *d* = 3.73, 95% CI [3.618, 4.103] (see Figure 1).

It is possible that the effect of voter group on post-referendum support for Brexit is fully mediated by the mechanisms of fear, uncertainty and control (Hayes, 2009). Hence, we ran a multiple mediated regression model in which these three mechanisms explained the effect of voting preferences on post-referendum support for Brexit. We further included the mechanisms of collective interests in order to test the alternative SIMSA proposition that post-referendum support for Brexit is more parsimoniously explained by group motives (Owuamalam et al., 2016a, 2018, 2019a). Finally, we included personal interests to check whether these were also influential. We ran this analysis in Mplus using 1,000 bootstrap resamples to examine the theorized indirect effects (see Table 3).

**Table 3:** Direct effects of voter preference and motives on support for Brexit (Study 1).

Effect of…	Mediator model				
	Uncertainty *ß* [95% CI]	Fear *ß* [95% CI]	Control *ß* [95% CI]	P_interest *ß* [95% CI]	C_interest *ß* [95% CI]
Voter preference	0.32 [0.204, 0.417]	−0.18 [−0.288, −0.085]	−0.05 [−0.155, 0.069]	0.35 [0.244, 0.445]	0.25 [0.155, 0.351]
	Dependent variable model				
	Support for Brexit *ß* [95% CI]				
Uncertainty	−0.02 [−0.074, 0.040]				
Fear	−0.04 [−0.103, 0.012]				
Control	0.05 [0.004, 0.102]				
Personal interest (P_interest)	−0.06 [−0.123, 0.000]				
Collective interest (C_interest)	0.09 [0.014, 0.170]				
Voter preference	−0.88 [−0.931, −0.825]				
*R* ^2^	0.79, *p* < 0.001				

Voter preference is coded 1 = system change (Brexit) voters, 2 = System supporting (remain) voters. This saturated model was generated in Mplus, and reported are standardized regression coefficients. Bootstrap resamples = 1,000.

Results revealed that only the mechanisms of personal and collective interests explained post-referendum support for Brexit among system supporters relative to system changers. For system supporting remain voters (relative to leave voters), personal interest explained significantly *reduced* support for the new post-referendum Brexit era (see Table 4), while collective interest explained significantly *increased* post-referendum support for Brexit (see Table 4). None of the SJT mechanisms of fear, uncertainty, and control maintenance explained post-referendum support for Brexit among system supporting voters (see Table 4).

**Table 4:** The indirect effect of voting preference on Post-Referendum Support for Brexit (Study 1), Post-Election Support for a Trump administration (Study 2).

*via*…	Study 1	Study 2
	Support for Brexit *ß* [95% *CI*]	Support for Trump *ß* [95% *CI*]
Uncertainty	−0.006 [−0.027, 0.012]	−0.023 [−0.047, −0.007]
Fear	0.008 [−0.001, 0.023]	0.006 [−0.006, 0.020]
Control maintenance	−0.002 [−0.013, 0.003]	0.001 [−0.007, 0.012]
Personal interest	−0.022 [−0.049, −0.001]	−0.003 [−0.019, 0.006]
Collective interest	0.023 [0.005, 0.049]	0.014 [0.001, 0.043]
System justification motive	−	−0.003 [−0.021, 0.014]

Standardized regression coefficients are reported. Bootstrap resamples = 1.000. Bootstrapped CIs are bias corrected (Hayes, 2017).

### Summary

Taken jointly, these analyses show that personal and collective interests (a) are more prominent reasons among system supporting Remain voters than among those Leave voters who clamored for system change, and that (b) group motives best explained post-referendum support for Brexit. In addition, Study 1 also raises questions about the system inevitability caveat that we derived from the SJT literature (see, e.g., Gaucher et al., 2010), in that after the referendum those who voted to remain should have scored higher (or equally) in their post-referendum support for Brexit compared to those who voted to leave, given the inevitability of Brexit. In other words, facing the inevitability of a new Brexit era, remain voters ought to have strongly embraced the “new system.” However, contrary to this SJT-based prediction, remain voters maintained their voting preference prior to the results being announced, and this outcome is more consistent with an identity-based account that accommodates the possibility of polarization or the hardening of political positions after the referendum (see Paicheler, 1979; Van Bavel and Pereira, 2018).

In short, these findings are more supportive of SIMSA’s position that group interests and identities provide a better explanation of system justification than does a separate system justification motive that operates independently of collective interests. They also suggest that SJT might be ill-equiped to explain system-related attitudes, and/or what the system might be with respect to the new political populism. However, Study 1 only assessed the proposed antecedents of the system justification motive; it did not assess the endorsement of the system justification motive itself. Also, Brexit is just one context, focused on a very specific policy issue and therefore perhaps not representative of other examples of the new political populism. Therefore, Study 2 examined another political context that is perhaps more representative of the new populism: the election of Trump.

## Study 2: The United States’s 2016 Presidential Election

Commentators have likened the 2016 United States election of the anti-establishment candidate Donald Trump to the populist Brexit movement in the United Kingdom (e.g., Witte, 2016). As with the Brexit context, the system justification enabling conditions of heightened system threat, dependency and inescapabilty were apparent in the current context (Jost and Hunyady, 2005; Kay and Zanna, 2009). For example, the election of Trump to office of the president threatened the status quo of not only the political establishment in Washington (with his “drain the swamp” campaign promise, see McGee, 2016), but also the health insurance system (i.e., Obamacare) on which many American citizens depended. Finally, the inescapability of a Trump administration should be apparent to system-supporting Hillary Clinton voters post-election, when results revealed that Trump had won, which was why the current data was collected after the election results announcement.

### Method

#### Sample Size and Participants

We used a similar sample size as in Study 1. However, of the 545 attempts on this survey *via* Prolific, only 289 cases were complete and useable based on the exclusion criteria that we used in Study 1. Of this number, 150 were men and 138 were women (1 did not indicate their gender). Participants (150 men, 138 women, and one non-disclosure; *M*_age_ = 36.02 years, *SD*_age_ = 13.90 years) resided in the United States and voted in the 2016 presidential election. Seventy-three percent were White, 14.5% were Black, and 12.1% were Latino. Participants received a pro-rata payment of US $6 per hour in exchange for completing the study questionnaire, which also included other measures unrelated to specific hypotheses tested here. We programmed our online survey software to collect equal numbers of cases for each group of voters in the 2–3 weeks following the 2016 United States election, and while the numbers in each group varied slightly after exclusions, this difference was negligible: Hillary Clinton voters (*n* = 150) and Donald Trump voters (*n* = 139), *X*^2^(1) = 0.42, *p* = 0.518.

#### Materials and Procedure

The materials and procedure in the current study were similar to Study 1 except that we also directly tapped the system justification motive using two items: “I voted the way I did…” (a) “because I want to *preserve* the existing political system,” and (b) “because the existing political systems function as they should.” As in Study 1, we measured SJT’s mechanisms of fear, uncertainty, and control maintenance. We also assessed personal and collective interests as in Study 1. All motives were measured on a seven-point scale (1 = *strongly disagree*, 7 = *strongly agree*).

We assessed participants’ trust in their government using the same three-item scale described in Study 1 (*α* = 0.739), and we measured combined annual household income as an indicator of social class (“Less than $10,000,” “$10,000–$19,999,” “$20,000–$29,999,” “$30,000–$39,999,” “$40,000–$49,999,” “$50,000–$59,999,” “$60,000–$69,999,” “$70,000–$79,999,” “$80,000–$89,999,” “$90,000–$99,999,” “$100,000–$149,999,” and “more than $150,000”). We included the same eight-item measure of personal sense of power that we described in Study 1 (1 = *strongly disagree*, 7 = *strongly agree*; *α* = 0.90).

Next, we measured participants’ post-election support for a Trump administration with a three-item scale that was similar to the one that we used in Study 1 in order to test SJT’s system-inevitability caveat: “I am pleased that America voted Donald Trump into the White House;” “the Obama Administration is a failed project and I support America’s decision to elect Donald Trump to the Presidency;” and “I would vote for Donald Trump if a second Presidential Election was opened to the public” (1 = strongly disagree, 7 = *strongly agree*, *α* = 0.97).

### Results and Discussion

As in Study 1, we checked whether those who voted for the establishment candidate (Hillary Clinton) were more likely to have greater trust in their government compared to those who voted for the change candidate (Donald Trump). Thus, we compared participants’ level of trust in their government across the two voter groups. Consistent with Study 1, system supporters (Clinton voters) reported greater trust in the prevailing government (*M* = 2.47, *SD* = 0.73) compared to system changers (*M* = 2.25, *SD* = 0.81), *t*(287) = 2.43, *p* = 0.016, Cohen’s *d* = 0.29, *SE* = 0.12, and 95% CI [0.054, 0.518].

The results of our correlation analysis of the key SJT mechanisms and those related to personal and group interests are shown in Table 1. Next, we examined whether the mechanisms proposed by SJT were more apparent for system supporters than for system changers (see Table 2). Contrary to SJT, the need to escape uncertainty was *less* (not more) prominent for system supporters compared to system changers, *t*(285.09) = 3.55, *p* < 0.001 (see Table 2). Corroborating the evidence in Study 1 and contrary to SJT, the need to maintain control over one’s life outcomes was no more prominent for system supporters than for system changers, *t*(283.10) = 0.07, *p* = 0.944 (see Table 2). Again, as in Study 1, group and personal interests were more prominent for system supporters than for system changers: group interests *t*(287) = 2.07, *p* = 0.039; personal interests *t*(287) = 2.48, *p* = 0.014 (see Table 2). As in Study 1, none of these effects were moderated by income-based social class (*p*s > 0.100) or by personal sense of power (*ps* > 0.220). Hence, high and low social class individuals’ support for the status quo were similarly motivated by personal and group interests.

#### Does the Effect of Voter Group on System Justification Motive Depend on Social Class/Power?

To answer this question, we performed a moderated regression analysis in which voter group (effect coded: −1 = system changers, 1 = system supporters) predicted the system justification motive conditional upon social class/power (centered around their means, Aiken and West, 1991). Considering the strong correlation between the two system justification items (see Table 1), and because both are often theorized to be part of a broader construct (Jost and Hunyady, 2005), we combined them to form a single index of system justification motive.

**Figure 2:**
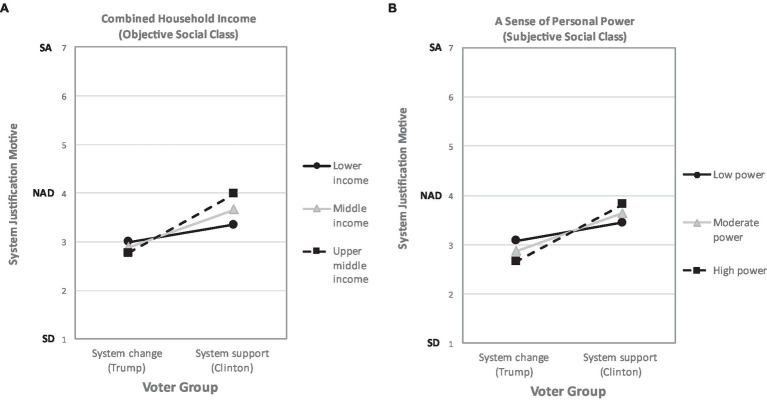
The effect of voter group on the system justification motive is qualified by indicators of objective **(A)** and subjective **(B)** social class. SA, strongly agree; NAD, neither agree nor disagree (unsure); and SD, strongly disagree.

Consistent with SJT, results revealed that system supporters disagreed with the system justification motive less strongly than system changers, *t*(287) = 4.05, *p* < 0.001 (see Table 2 for descriptive statistics). Results further revealed a significant voter group by social class interaction, *ß* = 0.13, *SE* = 0.06, *p* = 0.025, and 95% CI [0.017, 0.250]. However, contrary to the strong dissonance-inspired version of SJT, inspection of the simple slopes revealed that the voter group effect was restricted to the higher social class (*M* + 1SD; see Table 5 for simple slope estimates and Figure 2 for estimated means) and absent among the lower social class (*M* – 1SD; see Table 5 and Figure 2A). It is also possible to investigate these effects within each voter group (adjusting the alpha level downwards to 0.025 to account for multiple comparisons). This analysis corroborated the earlier one and showed—among system supporters—a positive (rather than negative) relationship between social class and system justification motive, *ß* = 0.10, *SE* = 0.04, *p* = 0.012, and 95% CI [0.022, 0.756]. This relationship was absent for system changers, *ß* = −0.04, *SE* = 0.04, *p* = 0.432, and 95% CI [−0.122, 0.052]. We repeated the same moderated regression analysis, this time substituting social class with a sense of personal power (see Table 5): the results were similar to those obtained using the social class index (*M* − 1SD, see Figure 2B; Table 5 for simple slopes).

**Table 5:** The moderating role of Objective and Subjective Social Status Indicators on the Effect of Voter Group on System Justification Motive.

Effects	Indicators of social class (and Disadvantage)	
	Objective social status (Social class)	Subjective social status (Sense of personal power)
	*ß* [95% *CI*]	*ß* [95% *CI*]
*Main and interactive effects of…*		
Voter group	0.39 [0.204, 0.574]	0.39 [0.199, 0.570]
Social status	0.11 [−0.075, 0.296]	−0.01 [−0.193, 0.179]
Voter group × Social status	0.21 [0.027, 0.400]	0.20 [0.013, 0.385]
*Simple slopes when social class is…*		
Low (*M* – 1*SD*)	0.18 [−0.089, 0.439]	0.19 [−0.077, 0.449]
Moderate (*M*)	0.39 [0.204, 0.574]	0.39 [0.199, 0.570]
High (*M* + 1*SD*)	0.60 [0.341, 0.864]	0.58 [0.321, 0.846]

Dependent variable in both models is the combined index of system justification motive. Social status is an umbrella expression that we have used to describe objective (income-based social class) and subjective (personal sense of power) social status.

#### Testing System-Inevitability Induced Support for Trump

**Figure 3:**
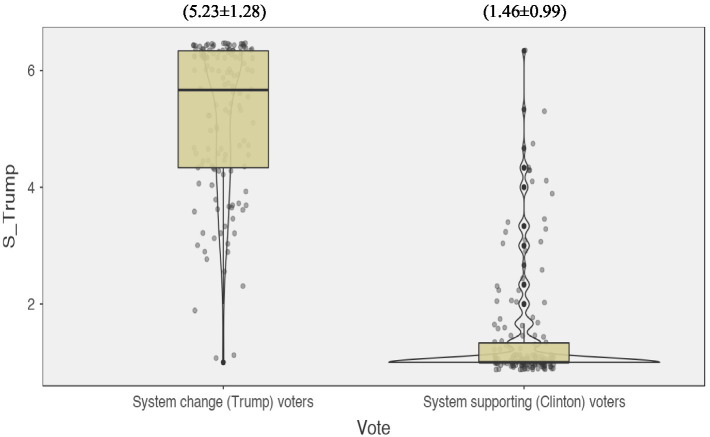
Violin plots for the distribution of scores within the system supporting vs. system change voting groups for post-election support for the new Trump era. Numbers above the plots = (Mean ± *SD*). S_Trump = support for Trump.

Corroborating the evidence in Study 1, and contrary to SJT’s system-inevitability caveat, results from an independent *t*-test revealed that post-election support for Trump was significantly *weaker* among system supporters than among system changers, *t*(258.68) = 28.12, *p* < 0.001, Cohen’s *d* = 3.31, 95% CI [3.504, 4.032] (see Figure 3).

As in Study 1, we followed up this simple descriptive analysis with the same multi-mediation regression model in which fear, uncertainty, control, personal, and collective motives, plus the system justification motive explained the effect of voter group on post-election support for a Trump administration. As before, we ran this analysis in Mplus using 1,000 bootstrap resamples to examine the theorized indirect effects (see Table 6).

**Table 6:** Direct effects of voter preference and motives on support for Trump (Study 2).

Effect of…	Mediator model					
	Uncertainty *ß* [95% *CI*]	Fear *ß* [95% *CI*]	Control *ß* [95% *CI*]	P_interest *ß* [95% *CI*]	C_interest *ß* [95% *CI*]	SJM *ß* [95% *CI*]
Voter preference	−0.21 [−0.332, −0.109]	0.18 [0.063,282]	−0.01 [−0.129, 0.100]	0.14 [0.034, 0.256]	0.12 [0.008, 0.241]	0.24 [0.118, 0.348]
Effects of…	Dependent variable model					
	Support for a Trump administration *ß* [95% CI]					
Uncertainty	0.11 [0.038, 0.177]					
Fear	0.03 [−0.036, 0.102]					
Control	−0.07 [−0.150, 0.003]					
Personal interest (P_interest)	−0.02 [−0.099, 0.040]					
Collective interest (C_interest)	0.12 [0.040, 0.195]					
System justification motive (SJM)	−0.01 [−0.084, 0.059]					
Voter preference	−0.84 [−0.882, −0.785]					
*R* ^2^	0.74, *p* < 0.001					

Voter preference is coded 1 = system change (Trump) voters, 2 = System supporting (Clinton) voters. Saturated model generated in Mplus using maximum likelihood (with bootstrap) estimation. Standardized regression coefficients are reported. Bootstrap resamples = 1.000.

Results revealed that the mechanisms of uncertainty avoidance reliably explained a *reduction* in support for Trump among system supporters relative to system changers (see Table 4). Also, corroborating the pattern of results from Study 1, collective interest explained a significant *increase* in post-election support for Trump among system supporters relative to system changers (see Table 4). None of the other SJT mechanisms of fear, control maintenance or the system justification motive itself explained post-election support for a Trump administration among system supporters (see Table 4). Although personal interest explained reduced post-election support for a Trump administration among system supporters (relative to system changers), this mediational effect was not reliably different from zero (*ß* = −0.003, see Table 4).

## General Discussion

An unresolved question in the debate between system justification and social identity scholars has been whether support for the status quo especially amongst the disadvantaged (e.g., losing voters) is due to collective interests or to a separate system justification motive. We addressed this question in the context of two real-world political events (Brexit and Trump’s election) by examining whether the system justification mechanisms of uncertainty avoidance, fear, and control maintenance were more apparent for (a) system supporters relative to those who rejected the system and sought social change, and (b) the disadvantaged lower class relative to the privileged class. We further explored the predictive potential of SJT’s system inevitability caveat, in order to test whether people relinquish their preferred systems once a new arrangement that sits at odds with their (collective) interests has been established (Gaucher et al., 2010).

In two studies, we found little consistent evidence for SJT’s proposal that uncertainty avoidance, fear, or the need to maintain control over one’s life predicted justification of the status quo. Consistent with SJT, Study 1 found that uncertainty avoidance was a significantly more prominent reason for system supporters (remain voters) compared to system changers (leave voters). However, in Study 2, uncertainty avoidance was a significantly *less* prominent reason for system supporters (Clinton voters) compared to system changers (Trump voters). Need to maintain control over one’s life outcomes did not differ significantly between system supporters and system changers in either study. Finally, fear was a *more* prominent reason for system supporting voters in Study 2, but a *less* prominent reason for them in Study 1.

Both studies found that personal and collective motives (a) were more consistently prominent for system supporters (i.e., supporters of the extant/prior system) than for system changers and (b) group interest operated as mediator of the relation between voter group and post-voting support for the imminent system. In relation to point “b,” in particular, the findings that system supporters were more likely to embrace the imminent system “because it was in the best interest of *my country*” aligns with SIMSA’s proposition that an awareness of interests that are connected to an inclusive identity can elicit an ingroup favoring system justification at the superordinate level of self-categorization (Owuamalam et al., 2018, 2019a,b; Caricati et al., 2021). That is, Remain/Clinton supporters may embrace the status quo because it serves the interest of their country to do so.

System justification theory predicts that personal and collective interests are most likely to motivate system justification among members of privileged groups and least likely to motivate system justification among members of disadvantaged groups. As we highlighted previously, Remain and Clinton supporters can be described as disadvantaged groups in the sense that they lost at the polls. With this in mind, we observed, contrary to SJT, that for these *ad-hoc* disadvantaged groups, collective interests were much stronger drivers of their voting preferences relative to the winning Leave/Trump voters. SJT’s proposition (i.e., the strong system justification thesis, Jost et al., 2004), would have anticipated the antecedents of the system motive to be more prominent for these political camps also (i.e. Remain and Clinton voters). In addition, the strong dissonance-based version of SJT also predicts that social class/sense of personal power should act as a moderator of voter group preferences when it comes to the mechanisms of uncertainty, fear, control maintenance, personal and collective interests. Contrary to this prediction, social class/sense of power did not qualify any effects involving personal and collective motives in either study. Hence, personal and collective interests seemed equally important for people at the lower and upper rungs of the social hierarchy (see also Owuamalam et al., 2016b; Owuamalam and Spears, 2020 for arguments against the strong dissonance-based version of SJT).

Finally, Study 2 found that although system supporters (Clinton voters) agreed with system justification more than system changers (Trump voters), this effect was restricted to participants who had a stronger sense of their personal power or who were high in income-based social class. These moderating effects of social class/subjective power further suggest that personal and collective interests underpin support for the status quo because people with a higher social class have vested personal and group interests in maintaining the status quo. A meta-analysis across the two studies also corroborated the conclusion that personal and collective motives were consistent and unambiguous drivers of support for the status quo (see Table 1).

### Limitation and Opportunities for Future Research

A key strength of the current investigation is that it examined some of the key arguments between SJT and SIMSA with regard to competing system and group motives as they played out in the real world. However, a disadvantage of this approach is that it precluded tight control over a number of potentially important moderator variables. In the view of Kay and Zanna (2009, p.162), two factors determine the potency of the system-inevitability caveat: “(i) perceptions of the extent to which the system is likely or unlikely to change (that is, its stability) and (ii) perceptions of the relative ease or difficulty with which the individual can exit the system and enter a new one (that is, its escapability).” Hence, SJT mechanisms should be most apparent when the system is seen as stable and people are unable to escape from it. This caveat may explain why the system motive was not particularly prominent in the context of the uncertainty that lingered over Brexit (post-referendum when the data were collected), although it does not explain the similar pattern of results that we obtained from the more *stable* American electoral context, in which a new system became *inevitable* once the election results were known.

It is also possible that the story might be different if the voting preferences were treated as the outcome rather than the predictor of the underlying motives that featured in our analysis. Hence, we re-ran our primary analysis, this time calculating a logistic regression function in which voting preference was the outcome, while all the motives were entered as predictors. Results from this analysis corroborated the ones that we reported earlier (see Appendix A, Figure A1): In Study 1, greater reports of personal and collective interests predicted reports of voting to remain in the EU, while fear and control anxieties predicted reports of voting to leave the EU. Identical patterns to those reported in our results section for Study 2 were also observed in a logistic regression re-analysis (see Appendix A, Figure A2). Again, we observed mixed evidence in Studies 1 and 2 with regard to the mechanism of seeking to avoid economic uncertainty: Supporting SJT, increased economic uncertainty was a potent predictor of self-reported vote to remain in the EU (Study 1). However, contrary to SJT, increased economic uncertainty predicted self-reported voting for social change (Trump) rather than system support (Study 2).

Another objection to the current findings could be that we used different measures of collective interest, personal interest, and system justification to those that are commonly used for these constructs in the literature. However, the use of other measures that are conceptually related to the traditional scale (e.g., for self/personal interest: personal and collective self-esteem; and for system justification: the general, economic, and political system justification measures) is arguably a key strength of the current investigation precisely because it addresses the question of convergent validity: the extent to which different operationalisations of an underlying construct yield the same result. For example, Jost et al. (2012, p. 200) recognized the importance of converging evidence in stating that:

to the extent that the various operationalizations of theoretical variables in different studies yield similar patterns of results, this research program as a whole will provide convergent evidence that general processes of system justification are at work and are not attributable to specific features of the groups or contexts under investigation

Although Azevedo et al. (2017) has examined system justification processes in the context of the 2016 United States presidential elections using a number of different measures of system justification, they did not examine (a) the roles that personal and group interests play or (b) how these processes might unfold across similar populist revolts elsewhere in Europe (e.g., Brexit). By closing these important gaps in the literature, and doing so with other operationalisations of the relevant constructs, the present analyses offer more complete and complementary insights. Nonetheless, future studies could aim to incorporate more widely used measures that are relevant to social identity and system justification to ascertain whether the pattern of results reported here replicate with such measures.

A further objection to the outcome of the current investigation, which was raised in the peer review process, is that:

right-wing contemporaneous populism is anchored on a notion of national nostalgia and the desire to go back in an attempt to return to a glorious past (Mudde, 2019). Therefore, *it is hard to catalog these movements as system-changing*, as they rather represent a reactionary approach that tries to go back to a past that involved essentially the same political system as that of today, *only based on more traditional, nationalist value* (Our emphasis in italics)

It is important to reiterate that we recognize the difficulty in characterising Brexit/Trump voters as system changers because their behaviour could be seen as support for traditional systems of the bygone era that may resemble aspects of the status-quo. However, there is at least one problem with this objection. *Reverting* to a bygone era, regardless of whether it shares certain things in common with the present, implies that some change (even if not absolute) must occur. Hence, it is accurate to catalog the populists’ preferences as “system-changing” because the action they took (i.e., voting) has a change implication for the *existing* system. That is, wishing to revert to a more traditional bygone system implies that the status quo must first be put aside or dismantled in some way (e.g., by “draining the swamp”), in order for its replacement with an older more nationalist system to occur. As we had stated in our opening preamble, the issue of where to locate the system (the present vs. the bygone era) is problematic only for SJT, because it is concerned with peoples’ attitudes toward “existing” rather than bygone societal arrangements (Jost and Banaji, 1994, p. 2; Jost, 2017, p. 74, Jost et al., 2004, p. 883; Jost, 2019, p. 266). In short, even if we were to accept that, for the populist voters, the system they had in mind was the bygone era, this would fall outside the explanatory remit of SJT, despite the recent attempt of Azevedo et al. (2017) to apply SJT in this context. Note, however, that this conundrum is absent under the social identity perspective because it accommodates the possibility of system change [*via* the social identity model of collective action (SIMCA), van Zomeren et al., 2008] and social stasis (*via* SIMSA; Owuamalam et al., 2018, 2019a,b).

A final objection to the present contribution is that we have acknowledged and measured the "system justification motivation" while the idea that such a motive exists–independent of personal and social identity needs–has received strong theoretical (e.g. Owuamalam et al., 2016b) and empirical (e.g., Owuamalam and Spears, 2020) opposition. It is important to note that our use of the term is in service of the system justification theory and, we would like to point out that SIMSA researchers have yet to acknowledge the existence of a separate system justification motivation that functions independently of personal and group motives. In terms of measurement, it is perhaps also informative to note that participants did not really agree with the two items that assessed the system motive in Study 2: Indeed, responses largely fell on the *disagree* end of the scale (i.e. below the neutral midpoint) on average.

## Conclusion

In light of the new political populism across Europe, North America and elsewhere, we examined whether a system justification perspective or a social identity model of system attitudes best explains the motivations of people who wanted to retain the existing order or to change it. Findings from our analyses suggest that the motivations for both camps of the political divide are best characterized as rooted in personal and collective interests rather than resulting from a separate system justification motivation (see also in this issue works by Caricati et al., 2021; Carvalho et al., 2021; Degner et al., 2021; Lönnqvist et al., 2021).

## Author’s Note

For the first time, we address the key issue concerning the underlying motivation for supporting societal systems sometimes found among society’s disadvantaged, from the perspectives of system justification theory and social identity model of system attitudes. We show that in the real world context of the populism movement that gripped the United Kingdom and United States in 2016 (in the wake of Brexit and the election of Donald Trump to the office of United States presidency) that personal and collective interests more parsimoniously explained people’s system support relative to motives rooted in the system.

## Data Availability Statement

The original contributions presented in the study are included in the article; further inquiries can be directed to the corresponding author.

## Ethics Statement

The studies involving human participants were reviewed and approved by University of Nottingham, Faculty of Science Ethics Committee. The patients/participants provided their digital informed consent to participate in these online studies. The studies were conducted in line with the guidelines set by the British Psychological Society.

## Author Contributions

All authors listed have made a substantial, direct, and intellectual contribution to the work and approved it for publication.

## Conflict of Interest

The authors declare that the research was conducted in the absence of any commercial or financial relationships that could be construed as a potential conflict of interest.

## Publisher’s Note

All claims expressed in this article are solely those of the authors and do not necessarily represent those of their affiliated organizations, or those of the publisher, the editors and the reviewers. Any product that may be evaluated in this article, or claim that may be made by its manufacturer, is not guaranteed or endorsed by the publisher.

## Figures and Tables

**Figure A:**
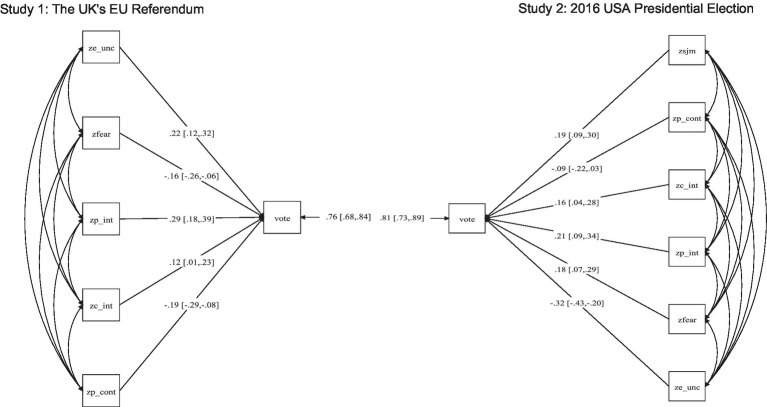
Logistic regression model with reasons for voting as predictors while reported voting preference (vote) is the outcome. Reported are standardized estimates are presented outside the parentheses, while the 95% CIs are presented within the parentheses. The analyses (and figures) were generated in Mplus version 7 (using maximum likelihood estimation). Variable Key: zc_unc = uncertainty; zfear = fear; zp_int = personal interests; zc_int = collective interest; zp_cont = need to maintain control; zsjm = system justification motive.

## Footnotes

^1^The studies reported in this paper were posted online *via* Prolific.ac with a link to Qualtrics where they were set-up. We *manually* screened participants in order to generate roughly equal numbers of Remain/Hillary Clinton and Leave/Donald Trump supporters, a screening options in Prolific.ac at the time. Therefore, those who clicked the study link and read the study information but then found out that they were not eligible were also recorded in Qualtrics as attempts. This resulted in many noncompletion hits. A paid research assistant who collated the data cleared out those non-completed attempts, and the analyses across both studies were based on cases that actually provided data.

^2^It is important to note that prominent SJT scholars have identified interests that are tied to the superordinate ingroup (e.g., country) as a group-relevant motive rather than a system motive (e.g., Kay et al., 2009, p. 428).
